# Impact of gasless vNOTES vs. traditional vNOTES on hemodynamic profiles and outcomes in patients with benign gynecological disease: study protocol of a randomized controlled trial

**DOI:** 10.1186/s12871-023-02322-7

**Published:** 2023-11-15

**Authors:** Kai Liu, Qinghua Huang, Yanjun Wang, Qianqian Zhang, Dan Feng, Zhaolin Gong, Jiaojiao Chen, Li He, Yu Cui, Yonghong Lin

**Affiliations:** 1 Department of Anesthesiology, School of Medicine, The Affiliated Hospital, UESTC Chengdu Women’s & Children’s Central Hospital, No.1617, Riyue Avenue, Qingyang District, Chengdu, 610091 China; 2 Department of Gynecology, School of Medicine, The Affiliated Hospital, UESTC Chengdu Women’s & Children’s Central Hospital, No.1617, Riyue Avenue, Qingyang District, Chengdu, 610091 China; 3 Department of Operating Room, School of Medicine, The Affiliated Hospital, UESTC Chengdu Women’s & Children’s Central Hospital, Chengdu, 610091 China

**Keywords:** Gasless vNOTES, Hemodynamic profiles, Benign gynecological disease, Randomized controlled trial

## Abstract

**Background:**

Literature regarding the advantages of gasless vNOTES is insufficient. The aim of our study is to compare gasless vNOTES vs. traditional vNOTES on hemodynamic profiles and outcomes in patients with benign gynecological disease. We hypothesize that compared with those in the traditional vNOTES group, hemodynamic profiles will be changed less during gasless vNOTES, while safety can be promised.

**Methods:**

This is a single-center, prospective, single-blind, randomized controlled clinical trial, which has been approved by the Institutional Review Board of Chengdu Women’s and Children’s Hospital on September 27, 2022. One hundred and twenty patients will be recruited and randomly assigned to either the traditional vNOTES group or the gasless vNOTES group in a 1:1 ratio. For patients allocated to the traditional vNOTES group, after insertion of one port through the vagina, CO_2_ gas is infused with a pressure of 12–14 mmHg; while for those allocated to the gasless vNOTES group, a special device is used as an abdominal wall-lifting device to facilitate gasless surgery. CO_2_ pneumoperitoneum will not be used during the whole gasless vNOTES procedure. The primary outcome is vital signs at different time points. The secondary outcomes include surgical conversion rate, duration of surgery and anesthesia, anesthetic consumption, intraoperative estimated blood loss, VAS and PONV scores at postoperative 2 h and 24 h, administration of vasopressor drugs from the beginning of general anesthesia induction to 15 min after endotracheal intubation, including times, dosage, and type, intraoperative and postoperative complications, time of first getting out of bed after surgery, and time of first eating after surgery, including light drink.

**Discussion:**

This is the first randomized controlled trial to compare the impacts of gasless vNOTES vs. traditional vNOTES on hemodynamic profiles and outcomes in patients with benign gynecological disease. If a favorable effect and safety of gasless vNOTES for hemodynamic profiles and outcomes in patients are shown, gasless vNOTES would be an optimal treatment option for patients with benign gynecological disease.

**Trial registration:**

The trial was registered at https://www.chictr.org.cn/showproj.html?proj=182441 with registration No. ChiCTR2200064779 on Oct 17, 2022.

**Supplementary Information:**

The online version contains supplementary material available at 10.1186/s12871-023-02322-7.

## Background

Since Transvaginal Natural Orifice Transluminal Endoscopic Surgery (vNOTES) was applied and reported in 2007 by Marescaux et al., this technique has been increasingly conducted in the field of gynecology, such as hysterectomy [1, 2], adnexectomy [3], and myomectomy [3, 4]. Published literature has proved that, compared to traditional laparoscopic surgery, vNOTES hysterectomy is associated with a lower pain score after surgery and a shorter length of hospital stay [1]. Due to its minimally invasive and without abdominal incisions, maximum aesthetics can be achieved.

During the routine vNOTES procedure, carbon dioxide (CO_2_) pneumoperitoneum is required. CO2 insufflation may induce hemodynamic instability and acid–base imbalance. A well-summarized review has reported that acidosis, inflammation, oxidative stress, and hypothermia may all be associated with CO_2_ pneumoperitoneum [5]. Moreover, in an animal study, the authors found that an inflammatory response was evoked during pneumoperitoneum [6]. Additionally, the transvaginal insertion of devices to establish CO_2_ pneumoperitoneum makes the already narrow surgical space even more crowded, further exacerbating the inconvenience of transvaginal operations [7]. In 2014, Chen et al. performed the first transvaginal appendectomy under gasless laparoscopy [7]. Subsequently, they reported that transvaginal salpingo-oophorectomy with gasless laparoscopy was successfully conducted in ten patients [8]. However, technical difficulties associated with gasless laparoscopy, such as limited abdominal space and poor surgical visualization restrict its application.

To date, literature regarding the advantages of gasless vNOTES is insufficient. Most studies related to gasless vNOTES are case series, which is not convincing enough. Thus, the aim of our study is to compare gasless vNOTES vs. traditional vNOTES on hemodynamic profiles and outcomes in patients with benign gynecological disease. The primary outcome is the changes in mean arterial pressure (MAP) and heart rate (HR) at different time points during the surgery. The secondary outcomes will be surgical conversion rate and complications. We hypothesize that compared with those in the traditional vNOTES group, hemodynamic profiles will be changed less during gasless vNOTES, while safety can be promised.

## Methods/design

### Study design, ethics, and trial registration

This is a single-center, prospective, single-blind, randomized controlled clinical trial. This study was approved by the Institutional Review Board of Chengdu Women’s and Children’s Hospital on September 27, 2022 [IRB approved number: 2022(112)]. The study protocol has been structured in according with the SPIRIT 2013 Statement [9] and CONSORT 2010 guidelines [10]. The trial was registered at https://www.chictr.org.cn/showproj.html?proj=182441 with registration No. ChiCTR2200064779 on Oct 17, 2022. The written informed consent will be obtained from all patients before enrollment in the study.

### Patient inclusion and exclusion criteria

#### Inclusion criteria

- Patients aged ≧18 years and ≦60 years;
- Body mass index (BMI) 18–25 kg/m^2^;
- Patients with benign gynecological disease, including adenomyosis of the uterus, uterine fibroids, ovarian cysts, tubal lesions, and other non-malignant gynecological diseases;
- Patients are scheduled to undergo vNOTES surgery, under general anesthesia;
- American Society of Anesthesiology (ASA) status I–III;
- Patients are willing to participate and provide signed written consents for the clinical study.

#### Exclusion criteria

The patients will be excluded if they meet the following criteria: (1) Patients without sexual activities before surgery; (2) The patient is pregnant or lactating; (3) The anticipated surgical duration is longer than 180 min, which will be judged by the surgeon; (4) Patients with a history of two or more cesarean sections, suspected rectovaginal endometriosis, suspected malignancy, or active lower genital tract infection; (5) Participating in other clinical studies within 3 months.

### Enrollment of participants and allocation

#### Consent to participate, enrollment of participants

Written informed consent by patients or their authorized surrogates will be required in this trial. Investigators who have been trained for the study procedures will screen potential candidates before surgery, according to the inclusion and exclusion criteria. The patients will be informed of the advantages, disadvantages, and potential risks of participating in the current study. The patients or their authorized clients should give written informed consent.

#### Allocation, Randomization, and blinding

**Fig. 1:**
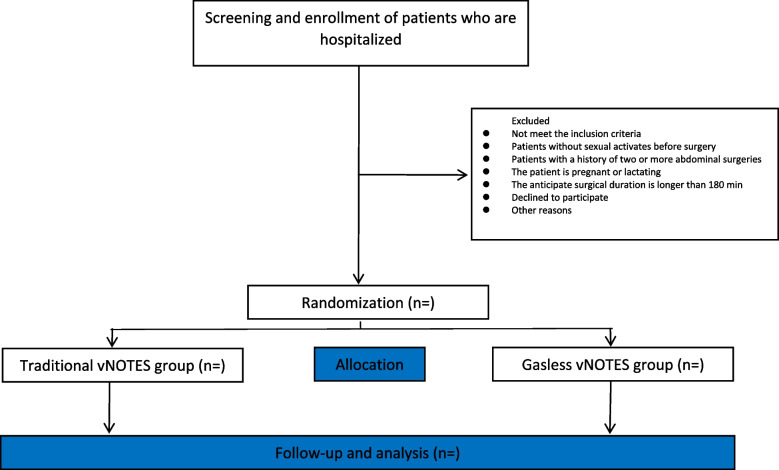
The study flow chart

This is a randomized, double-blind controlled clinical trial. One hundred and twenty patients will be recruited and randomly assigned to either the traditional vNOTES group or the gasless vNOTES group in a 1:1 ratio. A researcher from the anesthesiology department who is not involved in the current study will generate the randomized allocation number and distribute the allocated opaque envelope. The envelope will be delivered directly to the surgeon who is responsible for performing the surgery. The researchers involved in the postoperative assessment and the researchers analyzing the data are blinded to the groups’ assignment. When consent for trial participation is obtained from the participant, the physician will double-check the medical number, age, gender, and primary disease of the participant before unsealing the envelope to avoid reallocation and confirm their allocation. The study flow chart is presented in Fig. 1. The study process and evaluation are reported in Table 1. This study is in accordance with SPIRIT reporting guidelines, which are attached as an online Supplemental file 1.

**Table 1:**
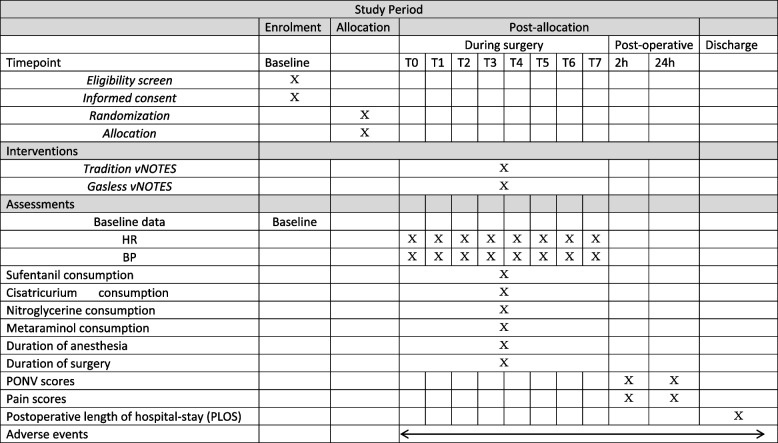
The experimental process and evaluation

### Interventions

Gasless vNOTES has been emergingly used recently, whereas the literature comparing the advantages between traditional vNOTES and gasless vNOTES is limited. Thus, we want to compare gasless vNOTES vs. traditional vNOTES on hemodynamic profiles and outcomes in patients with benign gynecological disease.

#### Intervention description

##### Anesthesia management and study intervention

The participants are continuously monitoring when they enter the operating room, including electrocardiograph (ECG), pulse oximetry, and non-invasive blood pressure. For the induction of the anesthesia, the patients are administered sulfentanyl (0.3 µg/kg), propofol (2 mg/kg), and cisatracurium (0.15 mg/kg) intravenously. Then, sevoflurane is adjusted for maintaining anesthesia and achieving a target BIS between 40–60 during the surgery. A single injection of sulfentanyl (0.1 µg/kg) and a bolus dose of cisatracurium (0.05 mg/kg) are permitted to be given independently during the surgery if necessary. When the surgery is completed, the patients are reversed with neostigmine 0.04 mg/kg and atropine 0.02 mg/kg if their TOF ratio is less than 0.9. Tracheal extubation is performed after the patient is fully awake and follows instructions.

If the patient’s MAP decreases > 20% compared to the baseline, the patient is given an intravenous injection of phenylephrine 40 µg. If the patient’s HR is detected to be less than 50 bpm, atropine will be given 0.3 mg per time. In the case when MAP increases to more than 30%, nitroglycerin 100 µg per time is administered.

##### Surgical technique

Since surgical proficiency may be one of the major potential influenced factors affecting the outcomes, all surgeries are performed by the specialist surgeons with more than 3 years of experience in performing vNOTES. All patients are placed in the lithotomy position. Standard vaginal and abdominal preparation is performed in all of our patients in case of conversion to transabdominal surgery. A Foley catheter is inserted to drain the urinary during surgery. The vaginal incision is made around the cervix.

For patients who allocate to the traditional vNOTES group, after insertion of one port through the vagina, CO_2_ gas is infused with a pressure of 12–14 mmHg. A 10-mm rigid 0°laparoscope is used, and energy devices to coagulate and remove the diseased tissue according to the location of the lesion. After the procedure is completed, vaginal cuff closure is done extracorporeally with continuous locking suture with 1–0 vicryl.

For patients who are allocated to the gasless vNOTES group, a special device is used as an abdominal wall-lifting device. One point is placed at the adnexa surface projection on the abdominal wall to establish the operating space, and the other point is placed under laparoscopic observation to facilitate gasless surgery. CO_2_ pneumoperitoneum will not be used during the whole procedure (Fig. 1).

After the surgery, patients will be transferred to the post-anesthesia care unit (PACU).

##### Criteria for discontinuing or modifying allocated interventions

Patients can quit the study at any time during the procedure, regardless of any reason. The investigators can discontinue patients’ participation if they are allergic to sedatives, have serious adverse events, are reluctant, or cannot be assessed after the procedure.

### Data collection

The following data will be collected during the study. To guarantee the study quality, all the investigators will be trained and pass the examinations. The trained investigator (YW) who will be blinded to the allocation will collect intraoperative data. KL, QH, and QZ will do the anesthesia procedures and collect intraoperative data, respectively; to avoid bias, they will not participate in postoperative data collection. All the statistical analyses will be performed by one of the corresponding authors (YC).

- Demographic data: Age, BMI, ASA status, clinical diagnosis, history of spontaneous delivery, abdominal surgery history, and surgical index.
- Intraoperative data: The MAP and HR prior to induction (T0), at the time point of intubation (T1), 10 min after tracheal intubation (T2), surgical incision immediately (T3), 10 min after surgical incision (T4), 10 min before the end of surgery (T5), at the end of surgery (T6), and at the time point of tracheal extubation (T7); duration of surgery; duration of anesthesia; the consumption of anesthetics (including sufentanil and cisatricurium); consumption of vasoactive drugs (including nitroglycerin and metaraminol); intraoperative estimated blood loss. Intraoperative complications include bowel, ureter, and bladder injury.
- Postoperative data
  *Postoperative pain assessment* All patients will be guided to use a 0 to 10 cm visual analogue Scale (VAS) to express the degree of pain. VAS scores are collected at the time of 2 h after surgery and 24 h after surgery. Additional analgesic medications are available at the patient’s request. Number of patients who have a requirement for rescue analgesia are recorded. Postoperative rescue analgesia can be provided by intravenous injection of 5 μg sufentanil or ketorolac 30 mg.
  *Postoperative adverse events *The incidence of postoperative nausea and vomiting (PONV) during postoperative 24 h are recorded. Nausea is referred to the patient with uncomfortable feeling in their stomach, and the severity of nausea is evaluated as follows*:* none = 0, mild = 1, moderate = 2*.* If there is gastric content vomited out, it is scored as 3. Rescue antiemetic could be offered to any patient who has a nausea score > 2. Postoperative complications include surgical site infection within 14 days, and unplanned secondary surgery related to this surgery.

### Data management and confidentiality

The CRF is designed according to our protocol and deposited in our special safe. Only the study team member who has permission from the principal investigator has access to this safe. Data will be entered into an electronic database and double-checked by the third investigator (DF). Any missing data or errors in the data will be summarized along with detailed descriptions and will be queried by checking the original forms. When the study is completed, the key to safety will be preserved by the principal investigator (YC) who is one of the corresponding authors. The detailed identification of the enrolled patients will not be reported in publications.

### Data monitoring

Based on previous publications, we will not establish a data monitoring committee due to this trial is small-scale and conducted at a single center [11]. If severe adverse events occur during the procedure, such as cardiac arrest or severe bleeding associated with procedures, the principal investigator has access to these interim results and makes the final decision to terminate the trial. And unblinding is permissible. Any modification to the protocol will be discussed and agreed to by the study investigators and will be subsequently submitted to the CRB for approval. We will also update the protocol at the Chinese Registry of Clinical Trials.

### Outcome measures

- 1. The primary outcome is vital signs at the different time points, including before anesthesia (baseline: T0), at endotracheal intubation (T1), 10 min after endotracheal intubation (T2), at the beginning of the operation (T3), 10 min after the operation (T4), 10 min before the end of the operation (T5), at the end of the operation (T6) and at the time of tracheal extubation (T7).
- 2. The second outcomes are collected as follows.
- ①Surgical conversion rate refers to the surgeon cannot complete the surgery in the scheduled mode of operation, and has to convert to open abdominal surgery or traditional multiport laparoscopic surgery, or single port laparoscopic surgery. For the patient who is allocated to the gasless vNOTES group, the surgeon requires the use of traditional vNOTES with CO_2_ pneumoperitoneum if they are unable to complete the surgery under gasless vNOTES, and this patient is considered as a surgical conversion. If the operation cannot be completed according to the scheduled surgical method, the surgical strategy can be changed as appropriate.
- ② Duration of surgery (from speculum placement to suturing the wound)
- ③ Duration of anesthesia (from anesthesia induction to endotracheal extubation)
- ④ The anesthetic consumption during the surgery, including sufentanil and cisatracurium
- ⑤ Intraoperative estimated blood loss
- ⑥ VAS and PONV scores at postoperative 2 h and 24 h.
- ⑦ Administration of vasopressor drugs from the beginning of general anesthesia induction to 15 min after endotracheal intubation, including times, dosage, and type.
- ⑧ Intraoperative and postoperative complications (e.g., bowel, ureter, and bladder injury, surgical site infection, and unplanned secondary surgery).
- ⑨ Time of first getting out of bed after surgery
- ⑩ Time of first eating after surgery after surgery, including light drink.

### Safety, adverse event reporting, and harms

Each safety issue will be defined when one or more of the following respective criteria are met. Intraoperative complications include bowel, ureter, and bladder injuries related to surgical technique. Postoperative complications referred to surgical site infection within 14 days, and unplanned secondary surgery associated with this surgery.

Adverse events are defined as all undesirable symptoms that may have a potential association with the investigational procedures. All adverse events are shared with the investigators and will be dealt with timely and appropriate. Serious adverse events related to this trial will be reported to the Chengdu Women’s and Children’s Central Hospital Certified Review Board (CRB) within 10 days.

### Sample size estimation

The sample size calculation was based on the changes in patients' blood pressure at the beginning of surgery. Since no similar studies have been conducted before, a pilot study was conducted previously. According to the results of the pilot study, in the traditional vNOTES group, 50% of patients had a greater than 20% change in blood pressure at the beginning of the CO_2_ pneumoperitoneum. Due to the different surgical strategies, CO_2_ pneumoperitoneum is not required in the gasless vNOTES group. We assume that the number of patients with a blood pressure change of more than 20% is less in the gasless vNOTES group than in the traditional vNOTES group. The 10% reduction in the gasless vNOTES group was considered to be statistically significant. A statistical power of 80% and a one-sided α significance level of 0.05 were used for power analysis with 1:1 allocation. A sample size of 49 patients in each arm was estimated. Considering a dropout rate of 20%, 60 patients for each group were enrolled.

### Statistical methods

Data will be analyzed by using R studio 4.2.2 for Windows (Armonk, NY, United States). Data from the primary outcome will be presented as mean ± standard deviation if normally distributed, and t-test will be conducted; otherwise, the data will be reported as median (IQR), and no-parametric tests will be performed. Categorical data will be summarized as frequencies (percentages) and compared between study groups using χ2 tests or Fisher exact tests, as appropriate. *P* value < 0.05 was considered significant.

## Discussion

This is the first randomized controlled trial to compare the impacts of gasless vNOTES vs. traditional vNOTES on hemodynamic profiles and outcomes in patients with benign gynecological disease. It is noted that pneumoperitoneum and carbon dioxide insufflations which are required during traditional vNOTES may lead to an increase in plasma catecholamine levels and plasma renin activity. Besides, the increased intra-abdominal pressure may lead to reduced thoracic and pulmonary compliance. All these changes result in an increase in heart rate, blood pressure, and reduced cardiac output. Moreover, a systematic review has well-summarized the “dark” disadvantages related to pneumoperitoneum effects, such as leading inflammatory response, inducing acidosis, producing reactive oxygen species, and adhesion development [5]. However, CO2 insufflation can provide a better visualization of the surgical field, which may shorten surgical duration and decrease surgical-related complications. If a favorable effect and safety of gasless vNOTES for hemodynamic profiles and outcomes in patients are shown, gasless vNOTES would be an optimal treatment option for patients with benign gynecological disease. Additionally, previous studies have proved that the use of vNOTES may be particularly effective and safe for selected populations, such as obese women, and women with large uteri [12, 13]. In our study, we may find that the application of gasless vNOTES is suitable for patients with abnormal cardiopulmonary functions.

## Trial status

Patient recruitment began on October 28, 2022. The trial was originally expected to end in December 2023, but unfortunately, we cannot complete it as expectation. The patient recruitment will be completed in March 2024 due to slow enrollment. The current protocol is version 1.3, dated September 01, 2022.

### Supplementary Information

**Additional file 1.**

** Additional file 2.**

## Data Availability

The datasets generated and/or analyzed during the current study are not publicly available but are available from the corresponding author on reasonable request.

## Abbreviations

VNOTES: Transvaginal Natural orifice transluminal endoscopic surgery
CO2: Carbon-dioxide
HR: Heart rate
BMI: Body mass index
ASA: American Society of Anesthesiology
MAP: Mean arterial pressure
PACU: Post anesthesia care unit
VAS: Visual analogue scale
PONV: Postoperative nausea and vomiting

## References

[1] Yan B, Miao HX, Wang Y, Xu JM, Lu XQ, He WH (2022). Hysterectomy by transvaginal natural orifice transluminal endoscopic surgery versus transumbilical laparoscopic single-site surgery: a single-center experience from East China. Biomed Res Int.

[2] Baron C, Netter A, Tourette C, Pivano A, Agostini A, Crochet P (2022). Initial experience with vNOTES hysterectomy for benign conditions in a French university hospital. Facts Views Vis Obgyn.

[3] Huang L, Feng D, Gu DX, Lin YH, Gong ZL, Liu DD (2022). Transvaginal natural orifice transluminal endoscopic surgery in gynecological procedure: experience of a women’s and children’s medical center from China. J Obstet Gynaecol Res.

[4] Baekelandt J (2018). Transvaginal natural-orifice transluminal endoscopic surgery: a new approach to myomectomy. Fertil Steril.

[5] Umano GR, Delehaye G, Noviello C, Papparella A (2021). The dark side of pneumoperitoneum and laparoscopy. Minim Invasive Surg.

[6] Papparella A, Noviello C, Ranucci S, Paciello O, Papparella S, De Biase D (2020). Pneumoperitoneum modifies serum and tissue CCL2-CCL5 expression in mice. JSLS.

[7] Chen YH, Wang DB, Tian Y, Wu SD (2014). Pure NOTES transvaginal appendectomy with gasless laparoscopy. J Surg Res.

[8] Liu T, Chen Y, Wang X (2020). Transvaginal salpingo-oophorectomy with gasless laparoscopy - an optional pure natural orifice transluminal endoscopic Surgery. Ginekol Pol.

[9] Chan AW, Tetzlaff JM, Gøtzsche PC, Altman DG, Mann H, Berlin JA (2013). SPIRIT 2013 explanation and elaboration: guidance for protocols of clinical trials. BMJ.

[10] Schulz KF, Altman DG, Moher D (2010). CONSORT 2010 statement: updated guidelines for reporting parallel group randomised trials. BMC Med.

[11] Minami T, Watanabe H, Kato T, Ikeda K, Ueno K, Matsuyama A (2023). Dexmedetomidine versus haloperidol for sedation of non-intubated patients with hyperactive delirium during the night in a high dependency unit: study protocol for an open-label, parallel-group, randomized controlled trial (DEX-HD trial). BMC Anesthesiol.

[12] Kaya C, Yıldız Ş, Alay İ, Aslan Ö, Aydıner İE, Yaşar L (2021). The comparison of surgical outcomes following laparoscopic hysterectomy and vNOTES hysterectomy in obese patients. J Invest Surg.

[13] Kaya C, Yıldız S, Alay I, Karakaş S, Durmuş U, Güraslan H (2021). Comparison of surgical outcomes of total laparoscopic hysterectomy and vNOTES hysterectomy for undescended enlarged uteri. J Invest Surg.

