# Supplementary material for: Impact of gasless vNOTES vs. traditional vNOTES on hemodynamic profiles and outcomes in patients with benign gynecological disease: study protocol of a randomized controlled trial
Source: BMC Anesthesiol. 2023 Nov 15;23:373. doi: 10.1186/s12871-023-02322-7 (PMC10648332; doi:10.1186/s12871-023-02322-7)
Supplement: Supplementary file 2 — Additional file 2. [file 12871_2023_2322_MOESM2_ESM.docx]

V 1.0 20220918

**Informed consent form**

Dear patient,

The doctor has determined that you need to perform a vNOTES. We will

invite you to participate in one study： Impact of gasless vNOTES vs. traditional vNOTES on hemodynamic profiles and outcomes in patients with benign gynecological disease: A prospective, randomized, controlled clinical study. This study was initiated by the Department of Anesthesiology of Chengdu Women and Children' s Central Hospital. This study protocol has been reviewed by the Ethics Committee of Chengdu Women’s and Children’s Central Hospital.

Before you decide whether to participate in this study, please read the following as carefully as possible. It can help you understand the study and why it was conducted, the procedures and duration of the study, the benefits,risks and discomfort you may have had after participating in the study. If you like, you can also discuss with your relatives and friends, or ask a doctor to explain and help you make decision.

**1. Research background and study objectives**

Transvaginal natural orifice endoscopic surgery (vNOTES) is the emerging

field of surgery that allows the surgeon to enter the peritoneal cavity

through a vaginal incision rather than an abdominal incision . In 2007,

vNOTES surgery was first reported in clinical practice, involving transvaginal endoscopic cholecystectomy. In 2012, Ahn et al applied it in a gynaecological patient, and two ovarian cysts were successfully removed by vNOTES without abdominal incision. Theoretically, vNOTES can reduce postoperative pain, accelerate postoperative recovery, reduce postoperative complications, and provide a scar less cosmetic effect. These advantages are also demonstrated inclinical practice. In 2015, Wang et al reported that the vNOTES group had shorter operative time, estimated blood loss (EBL), transfusion oxygen requirement, and postoperative hospital stay compared with traditional laparoscopic hysterectomy.

Although the advantages are obvious, vNotes, like conventional laparoscopy,

relies on CO 2 pneumoperitoneum to provide adequate visual field exposure. For adequate surgical space and good visualization, pneumoperitoneum is required. Theoretically, the exposure quality of pneumoperitoneum is increases, the higher the pneumoperitoneum pressure, the greater the impact on the respiratory and circulatory systems, which may cause huge intraoperative hemodynamic fluctuations, and the high incidence of postoperative shoulder pain, which is not conducive to patient recovery.

Therefore, we intend to compare the difference between the hemodynamic effects of conventional vNOTES and free vNOTES intraoperatively by conducting a prospective randomized controlled clinical study. We hypothesized that vNOTES without pneumoperitoneum is more conducive to intraoperative hemodynamic stability and will facilitate postoperative recovery.

**1.2 Purpose of this study**

The aim of our study is to compare gasless vNOTES vs. traditional vNOTES on hemodynamic profiles and outcomes in patients with benign gynecological disease. The primary outcome is the changes in mean arterial pressure (MAP) and heart rate (HR) at the different time points during the surgery. The secondary outcomes will be surgical conversion rate and complications.

**1.3 Study participants and the number of expected participants**

**Inclusion criteria**

- Patients age ≧18 years and ≦60 years;
- Body mass index (BMI) 18-25 kg/m2;
- Patients with benign gynecological disease;
- Patients are scheduled to undergo vNOTES surgery, under general anesthesia;
- American Society of Anesthesiology (ASA) status I–III;
- Patients are willing to participate and providing a signed written consents for the clinical study.

**Exclusion criteria**

The patients will be excluded if they meet the following criteria: (1) Patients without sexual activates before surgery; (2) The patient is pregnant or lactating; (3) The anticipate surgical duration is longer than 180 min; (4) Patients with a history of two or more cesarean sections, suspected rectovaginal endometriosis, suspected malignancy, or active lower genital tract infection; (5) Participating in other clinical studies within 3 months.

Finally， 120 patients will be enrolled.

**3. what will you need to do if you participate in the study?**

Before you are enrolled in the study, the doctor will ask, record your medical history, and evaluate whether you can get into the study. If you are a qualified participant, you may voluntarily participate in the study and sign the informed consent form. If you do not want to participate in the study, we will follow your wishes.

If you volunteer to participate in the study, you will follow the following steps :

1. Randomization will be carried out according to the protocol of the selected groups.
2. Your clinical data during hospitalization will be collected.
3. Other matters requiring your cooperation: complete the questionnaire.

**4. Possible benefits of participation in the study**

This study will further clarify compared with those in traditional vNOTES group, hemodynamic profiles will be changed less during gasless vNOTES, while the safety can be promise.

1. **Possible adverse reactions, risks, discomfort and inconvenience in the study**

The surgeon cannot complete the surgery as the scheduled mode of operation, and have to convert to open abdominal surgery or traditional multiport laparoscopic surgery or single port aparoscopic surgery.

1. **Related expenses**

There is no additional fee.

1. **Confidentiality of personal information**

The CRF is designed according to our protocol and deposited in our special safe. Only the study team member who have permission from the principle investigator has access to this safe. Data will be entered into an electronic database and double-checked by the third investigator (DF). Any missing data or errors in the data will be summarized along with detailed descriptions and will be queried by checking the original forms. When the study is completed, the key of safe will be preserved by the principle investigator (YC) who is one of the corresponding author. The detailed identification of the enrolled patients will not be reported in publications.

1. **How do you get more information?**

You can ask any questions about this study at any time and the principle investigator will answer them accordingly.If there is any important new information during the study that may affect your willingness to continue participating in the study,your doctor will in form you in time.

1. **You can voluntarily choose to participate in the study and withdraw from the study**

Whether you will participate in the study is entirely dependent on your wishes. You may refuse to participate in the study or withdraw from the study at any time during the study , which will not affect your relationship with your doctor or the loss of your medical or other benefits.

For your best interest, your doctor or investigator may suspend you

at any time during the course of the study.

1. **Related expenses**

There is no additional fee.

1. **Confidentiality of personal information**

The CRF is designed according to our protocol and deposited in our special safe. Only the study team member who have permission from the principle investigator has access to this safe. Data will be entered into an electronic database and double-checked by the third investigator (DF). Any missing data or errors in the data will be summarized along with detailed descriptions and will be queried by checking the original forms. When the study is completed, the key of safe will be preserved by the principle investigator (YC) who is one of the corresponding author. The detailed identification of the enrolled patients will not be reported in publications.

- I can consult my doctor for more information.
- I can withdraw from this study at any time without discrimination or retaliation, and my medical treatment and interests will not be affected.
- I am also aware that if I withdraw from the study, especially when I withdraw from the study due to drug reasons, if I tell the doctor about the changes in my condition and complete the corresponding physical examination and physical and chemical examination, it will be very beneficial to the whole study.
- If I need to take any other medication due to the change in my condition, I will ask my doctor for advice in advance or tell him the truth afterwards.
- I agree with the ethics committee of the drug Authority or the sponsor representative to access my study data.
- I will obtain a copy of the signed and dated informed consent form.
- I confirm the details of the trial , including its authority and possible benefits and risks.
- Finally, I decided to agree to participate in this study, and I promised to follow the doctor' s advice as much as possible.

Signature of the patient or his family member:______________

Relationship with patients:_______________

Contact number: ______________ Date ___________

Doctor’s signature: __________ Date: _______

Phone number:_____________
